# Implementation of a Physician Assistant Emergency Medicine Residency Within a Physician Residency

**DOI:** 10.5811/westjem.2020.11.49052

**Published:** 2020-12-14

**Authors:** Alina Tsyrulnik, Katja Goldflam, Ryan Coughlin, Ambrose H. Wong, Jessica M. Ray, Jessica Bod, Sharon Chekijian, David Della-Giustina

**Affiliations:** Yale University School of Medicine, Department of Emergency Medicine, New Haven, Connecticut

## Abstract

Physician assistants (PA) are an important part of emergency department healthcare delivery and are increasingly seeking specialty-specific postgraduate training. Our goal was to pilot the implementation of a PA postgraduate program within an existing physician residency program and produce emergency medicine-PA (EM-PA) graduates of comparable skill to their physician counterparts who have received the equivalent length of EM residency training to date (evaluated at the end of first year of EM training).

The curriculum was based on the Society for Emergency Medicine Physician Assistants (SEMPA) recommendations with a special focus on side-by-side training with EM resident physicians. In reviewing the program, the authors examined faculty evaluations, as well as procedure and ultrasound experience that the trainees received. We found comparable evaluations between first-year EM-PA and physician trainee cohorts. This program serves as a pilot study to demonstrate the feasibility of collocating clinical and didactic programming for physicians and EM-PAs during their postgraduate training. This brief innovation report outlines the logistics of the clinical and didactic curriculum and provides a summary of outcomes evaluated.

## BACKGROUND

Physician assistants (PA) are an important part of the modern emergency department (ED), facilitating the practice of emergency physicians and serving as independent providers of patient care.^1^,^2^ Traditionally, PA education relies on experience gained in clinical rotations and postgraduate “on-the-job” practice. Increasing ED patient volumes and acuity burden providers and the healthcare system,^3^ compelling EDs to hire PAs to assist in providing emergency care. The breadth of knowledge required to manage diverse and high-acuity patients in EDs has led to the development of EM-PA residencies.

Research shows that collaborative healthcare teams decrease cost, improve patient satisfaction, and reduce morbidity and mortality through enhanced patient safety and reduction in errors. At the same time, interprofessional teams improve overall provider satisfaction and professional relationships.^4^ To promote this team-based approach in clinical care, the Institute of Medicine recommends an interprofessional model in healthcare education.^4^ Interprofessional education is rooted in situated learning theory, where a community of practice helps foster collaborative learning across professional silos.^5^ Medical education has increasingly focused on such interprofessional team development.^6^ In 2009, several medical profession education governing bodies, including the Association of American Medical Colleges, created the Interprofessional Education Collaborative to encourage and promote interprofessional learning experiences.^7^ The model of PA residency education presented here similarly applies this interprofessional framework.

The United States currently has 49 clinical postgraduate EM-PA training programs.^8^ There are diverse durations, curricula, geographic and demographic clinical settings (academic vs community), patient populations, and volume. Most importantly, the focus and goals of these programs vary.^9^

## OBJECTIVES

The goal of the program presented here was to pilot an implementation of PA postgraduate training embedded within the existing educational infrastructure of an EM residency program. Both PA and physician trainees engage in identical clinical and academic responsibilities together. Through this unique approach of collocating both trainee groups within the same educational milieu, EM-PA residents engage in identical clinical and academic activities as their physician resident counterparts.

## CURRICULAR DESIGN

This program was established at a Level I trauma, quaternary care referral center with an existing four-year physician residency consisting of 15 physician residents and two PA residents per class. The logistics of recruiting and credentialing of PA providers were similar to that of hiring non-resident PA providers and was based on institutional guidelines as well as best practices described in literature.^10^ The university institutional review board approved our work as an exempted study.

**Figure 1:**
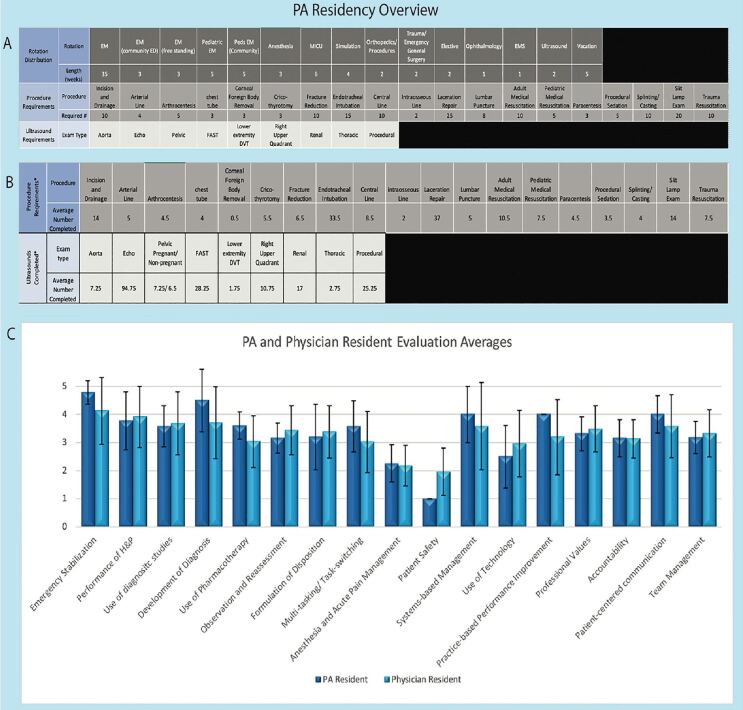
Overview of emergency medicine physician assistant residency. A. Format of the physician assistant (PA) residency: rotation description and length; procedure requirements; ultrasound requirements. B. Average (3 years of trainees) of procedures and ultrasounds completed by PA residents. C. PA and physician resident evaluations averages (and standard deviation) on 5-point Likert scale evaluated by attending physicians (average of 3 years of trainees). The evaluations are of EM-PA and EM-physician residents in their first year of EM residency training. *PA*, Physician Assistant; *EM*, emergency medicine; *EMS*, emergency medical services; *FAST*, focused assessment with sonography for trauma; *DVT*, deep vein thrombosis; *H&P*, history and physical.

Following the 2012 Society for Emergency Medicine Physician Assistants (SEMPA) recommendations,^11^ we established the EM-PA residency in 2015 as an 18-month program consisting of one- to four-week rotations (Figure 1A). All rotations were identical to the physician residency with the exception of one additional, four-week rotation at an affiliated freestanding ED for the PAs. The didactic curriculum included lectures, immersive simulation, reading assignments with associated quizzes, and an “EM Fundamentals” program.^12^ EM-PA residents completed a Rosh Review mock in-training examination, mock oral board examinations, cadaver lab, and airway training, and were members of quarterly wellness team meetings. Both trainee groups participated in all didactic, clinical, and residency-run social experiences together.

During all rotations, EM-PA residents functioned as primary team members. They worked alongside attending physicians with the same responsibilities as their EM postgraduate year 1 (PGY-1) (for the first 12 months of EM-PA training) or PGY-2 (for months 12–18 of EM-PA training) physician counterparts who were at the same point in their EM training. EM-PA residents also maintained procedure and ultrasound logs (Figure 1B).

## IMPACT/ EFFECTIVENESS

The program’s effectiveness and breadth of PA training was reflected in a number of parameters, including evaluations and procedure and ultrasound logs. We assessed for program feasibility by filling of available trainee positions in subsequent years, and retention of graduating EM-PAs in the field of EM immediately upon program completion.

Resident evaluations were collected at the end of the first year of EM training via an online software tool (Qualtrics XM, Provo UT) implemented in 2016 for both trainee groups.^13^ The tool uses a 5-point Likert scale to measure proficiency in EM-specific competencies that are loosely based on the EM Milestones.^14^ Means and standard deviations show similar scores for faculty evaluation of first-year physician and EM-PA residents’ performance during ED rotations between July 2017–July 2020 (Figure 1C). Limitations in these evaluation results include the fact that the evaluation tool randomly selects two questions, leading to inconsistent numbers of attending physician evaluations of various competencies. Further, each year only consisted of two PA trainees, thus limiting the ability to draw statistically significant conclusions. However, a descriptive similarity in scores can be observed.

PA residents had experience in all procedures and ultrasounds expected (Figure 1B). Similar to physician residents, PA residents performed some of the rare procedures, such as cricothyrotomy, in a cadaver lab or at the simulation center. It is important to note that the use of faculty evaluations, as well as procedure and ultrasound numbers, are only some of the many means of comparing the two trainee groups. Future investigation would need to aim at comparing other educational outcome measures.

Since its inception, the two positions available in the PA residency were filled each year. In the current academic year 2020–2021, the program increased to four positions per year. Upon graduation, all PA residents secured clinical positions in EM.

Several EM-PA residencies exist, with prior publications outlining best practices in the logistics of some of these programs.^10^ Although a recent cross-sectional study of EM-PA residencies identified that 93% of programs were in institutions that also had an EM residency program, it is unclear how the educational programs of their PA residents interacted with their respective MD residents.^2^ The aim here was to describe an EM-PA residency collocated and embedded within an existing physician residency and demonstrate the feasibility of this interprofessional approach to EM-PA postgraduate training.

It is important to note that the PA residency is 18 months long, in accordance with SEMPA recommendations. Graduates’ knowledge is not exhaustive, and on-the-job training after residency remains critical. The program, as designed, is not meant to provide PA trainees with the same level of training as EM residents upon residency completion. For that reason, the evaluation comparisons provided here are at the PGY-1 level. Finally, although there is much evidence supporting the benefits for integrated interprofessional education^4^ for enhancing teamwork and communication skills, this endpoint was not evaluated.

The structure of this program can be replicated across other EDs. Investigation should continue in evaluating the efficacy of this approach with higher numbers of graduates and more years of follow-up. Analyses of team management and communication skills as outcomes of such programs is needed. Although the authors intend to formally explore how the PA and physician residents feel about this interprofessional approach, the anecdotal response of both groups of learners has been predominantly positive. Despite the fact that the addition of PA residents has increased the number of learners in the department, our faculty, patient, and procedure numbers are able to support the additional trainees. In the authors’ experience, through informal observations and discussions with the residents, the fact that both groups share orientation, didactics, and social events from the moment they start residency, has contributed to their positive experience in clinical teamwork and colocation in the ED. From early experience, it stands to reason that the combination of adequate patient, procedure, and supervision availability in the clinical setting combined with outside educational experiences (didactics, simulation, reading assignments) and social events, allowed these two groups of learners to achieve a positive learning experience. With further study and evaluation, training PAs and physicians together clinically and in the classroom may prove effective in graduating well-trained, extremely competent and highly recruited EM-PAs.
